# Prediction of Monomeric and Dimeric Structures of CYP102A1 Using AlphaFold2 and AlphaFold Multimer and Assessment of Point Mutation Effect on the Efficiency of Intra- and Interprotein Electron Transfer

**DOI:** 10.3390/molecules27041386

**Published:** 2022-02-18

**Authors:** Yuri D. Ivanov, Amir Taldaev, Andrey V. Lisitsa, Elena A. Ponomarenko, Alexander I. Archakov

**Affiliations:** 1 Laboratory of Nanobiotechnology, Institute of Biomedical Chemistry, Pogodinskaya St. 10/8, 119121 Moscow, Russia; yurii.ivanov.nata@gmail.com (Y.D.I.); lisitsa060@gmail.com (A.V.L.); 2463731@gmail.com (E.A.P.); alexander.archakov@ibmc.msk.ru (A.I.A.); 2 Laboratory of Shock Wave Impacts, Joint Institute for High Temperatures of the Russian Academy of Sciences, Izhorskaya St. 13 Bd.2, 125412 Moscow, Russia; 3 Department of Chemistry, Sechenov First Moscow State Medical University (Sechenov University), Trubetskaya St. 8/2, 119991 Moscow, Russia

**Keywords:** cytochrome P450, CYP102A1, P450 BM3, AlphaFold, protein dimer prediction

## Abstract

The three-dimensional structure of monomers and homodimers of CYP102A1/WT (wild-type) proteins and their A83F and A83I mutant forms was predicted using the AlphaFold2 (AF2) and AlphaFold Multimer (AFMultimer) programs, which were compared with the rate constants of hydroxylation reactions of these enzyme forms to determine the efficiency of intra- and interprotein electron transport in the CYP102A1 hydroxylase system. The electron transfer rate constants (*k*_et_), which determine the rate of indole hydroxylation by the CYP102A1 system, were calculated based on the distances (R) between donor-acceptor prosthetic groups (PG) FAD→FMN→HEME of these proteins using factor β, which describes an exponential decay from R the speed of electron transport (ET) according to the tunnelling mechanism. It was shown that the structure of monomers in the homodimer, calculated using the AlpfaFold Multimer program, is in good agreement with the experimental structures of globular domains (HEME-, FMN-, and FAD-domains) in CYP102A1/WT obtained by X-ray structural analysis, and the structure of isolated monomers predicted in AF2 does not coincide with the structure of monomers in the homodimer, although a high level of similarity in individual domains remains. The structures of monomers and homodimers of A83F and A83I mutants were also calculated, and their structures were compared with the wild-type protein. Significant differences in the structure of all isolated monomers with respect to the structures of monomers in homodimers were also found for them, and at the same time, insignificant differences were revealed for all homodimers. Comparative analysis for CYP102A1/WT between the calculated intra- and interprotein distances FAD→FMN→HEME and the rate constants of hydroxylation in these proteins showed that the distance between prosthetic groups both in the monomer and in the dimer allows the implementation of electron transfer between PGs, which is consistent with experimental literature data about *k*_cat_. For the mutant form of monomer A83I, an increase in the distance between PGs was obtained, which can restrict electron transportation compared to WT; however, for the dimer of this protein, a decrease in the distance between PGs was observed compared to the WT form, which can lead to an increase in the electron transfer rate constant and, accordingly, *k*_cat_. For the monomer and homodimer of the A83F mutant, the calculations showed an increase in the distance between the PGs compared to the WT form, which should have led to a decrease in the electron transfer rate, but at the same time, for the homodimer, the approach of the aromatic group F262 with heme can speed up transportation for this form and, accordingly, the rate of hydroxylation.

## 1. Introduction

CYP102A1 belongs to the cytochrome P450 superfamily of enzymes. Cytochrome P450 plays an important role in the body, as it is involved in the metabolism of both endogenous and exogenous substrates, including the detoxification of poisons and drugs [1].

Unlike other types of cytochrome P450, CYP102A1 is self-sufficient. It contains heme and two flavin domains (FAD and FMN), which are linked by a single polypeptide chain. The electron donor in this system is NADPH [2]. The simplest method of electron transportation in this protein would be a way when, in this system, electron transfer occurs within one polypeptide chain sequentially from FAD to FMN and then to heme (FAD→FMN→HEME electron transport pathway) of the enzyme. However, according to the mechanism discussed in the literature [3], electron transport can occur in the dimeric complex. In fact, these two mechanisms of electron transfer, both in a monomeric form, where the activity towards the substrate (lauric acid) was shown at a level of 10 s^−1^, and in a dimeric one, for which the activity was of the same order of magnitude (50 s^−1^), did not exclude but rather complemented each other [4].

The struggle to understand the structural organization of the electron transport system, even the most seemingly simple CYP102A1 system, was caused by the fact that there were no experimental studies with a high spatial resolution (up to 1 Å) or opportunities for predicting the structure of this enzyme theoretically without considering a large number of experimental studies to clarify its molecular organization. It should be noted that the complete structure of CYP102A1 obtained by X-ray diffraction analysis is still unknown. Only the structure of the truncated protein was obtained, i.e., the CYP102A1 fragment containing the heme and FMN domains [5].

However, it recently became possible to resolve the structure of proteins employing cryoelectron microscopy (cryoEM) with a resolution of 1.15 Å [6], but as for CYP102A1, EM images of this protein were obtained with a resolution of 6–8 Å only for the mutant form CYP102A1/A83F, which was presented only as a dimer [3] since the monomer cannot be visualized by this method. Unfortunately, this structure is published in EMDB only in the form of an electron microscopy density map without specifying the positions of individual atoms, which makes it difficult for researchers who do not have the skills to work with cryoEM to work with the structure. It should also be noted that, despite this, cryoEM for the heme fragment dimer complex in this study was presented with an error of 0.6 Å relative to the XRD data, which provides fairly good accuracy in determining the protein structure.

Recently, a unique opportunity appeared to predict not only the structure of the enzyme, but also its complexes for water-soluble proteins as well as membrane ones. A possible solution to this problem would be the most adequate when using the AlphaFold2 (AF2) program to predict the structure of individual monomeric proteins [7] and AlphaFold Multimer (AFMultimer) to predict protein complexes [8].

In the presented work, the calculation of the distances between PGs in the electron transport enzyme CYP102A1 was possible due to the implementation of these programs. It is known that point mutations in this enzyme lead to a change in the hydroxylation rate constants for the A83F and A83I forms [9]. Therefore, a comparative analysis was carried out between the obtained PG distances in WT (wild-type) and mutants and the hydroxylation rate constants in order to reveal the mechanism of electron transport in these enzymes.

The rate constant of hydroxylation is determined by the rate of electron transport between donor-acceptor groups in a protein. According to the latest concepts, ET in proteins [10] between donor and acceptor groups can be simplified in terms of the mechanism of tunnelling electron transfer coupled with the rearrangement of the nuclear system and thermal dissociation of a part of the electron energy which consists of two parts:

W = W_0_ + W_1_e (−ћω/kT), (1)

where W_0_—probability of sub-barrier, temperature-independent tunnelling from lower vibrational levels; W_1_—probability of an over-barrier activation process; W_0_ << W_1_, ћω—vibrational quantum energy required to activate the process.

The over-barrier activation process determines the thermal activation energy for the molecule state transition to the upper vibrational levels of the initial state, where the coordinates of the nuclei are in the region of intersection of the potential energy of the initial and final states.

As can be seen from Equation (1), the probability of electron transport can be described by a term that does not depend on temperature and a term that depends on temperature, i.e., this process is quite complicated.

According to the theory of the tunnelling mechanism of electron transfer by Marcus, in the approximation of a non-adiabatic process, the dependence of the probability of electron transport on the distance, which determines the rate constant of electron transfer, can be represented in the form [10]:

W = A_0_e (−βR), (2)

where R—distance between donor and electron acceptor, β (describes the exponential decay of the electron transport rate)—characterises the degree of delocalization of the electron shells of the donor and acceptor centers.

Within the framework of this model (see Equation (2)), it can be concluded that electron transport should be observed most efficiently at short distances between the donor and acceptor. At the same time, a contribution of thermal activation to the processes of electron transportation determines the efficiency of the overlap of the energy levels of the initial and final states of the donor-acceptor pair. Therefore, the processes of electron transportation in homodimer and monomer are rather difficult to describe.

AF2 [7] and AFMultimer [8] programs were adapted to predict the structures of CYP102A1/WT and its dimeric complex. It was shown that the root-mean-square deviation (RMSD) of individual globular domains and experimental structures obtained by X-ray diffraction analysis did not exceed 0.9 Å, which is a good confirmation of the adequacy of the AF2 program. The structure of the full-size monomer CYP102A1/WT was calculated. Comparison of the prediction results for AF2 and AFM in the case of the full-length protein showed that the structure of the full-length protein is very different (RMSD 27.5 Å). That is, these three domains are not rigidly oriented in the protein globule. The accuracy provided by the AF2 program is at the level of the coincidence accuracy of the electron microscopy data for the dimer of the mutated form CYP102A1/A83F, which is about 1.0 Å [3].

It was shown that, in the case of their monomeric forms, there are significant differences in their structural organization of the mutual arrangement of FAD, FMN, and heme domains. In the case of dimeric forms, these differences were insignificant. Similar studies with the same results obtained were carried out for the CYP102A1 mutant forms A83F and A83I.

The distances between the prosthetic groups of both WT and CYP102A1 mutant forms were calculated from the obtained structures of both monomers and dimers. The relations between the distances between the prosthetic groups CYP102A1 and *k*_cat_ of indole hydroxylation were discussed; consequently, the following conclusions were drawn. For the monomeric form of WT, the limiting link for the ET reaction in the FAD→FMN→HEME path was a distance of 28 Å between FAD and FMN, which determined the ET constant of about 0.3 s^−1^. For the dimeric form, the limiting unit for ET was the same distance of 30 Å, but not between FAD and FMN, but between HEME1 and FMN1 in monomer 1 of the homodimer (as a result of structural rearrangement upon dimerization), which results in *k*_et_ = 0.03 s^−1^, which is commensurate with *k*_cat_ for indole. For the A83I form of the monomer, the limiting distance of 32 Å between FAD and FMN resulted in a very low value for the ET constant of about 0.004 s^−1^. For the homodimer of this form, the limiting distance between HEME2 and FMN2 in monomer 2 of the homodimer was at the level of 20 Å, which led to an increase in *k*_et_, compared to the WT form, in accordance with the literature data on *k*_cat_ [9]. For monomer A83F, the limiting distance for the ET reaction was the distance between FMN and heme, which was 40 Å. This did not allow the implementation of effective ET. However, for the homodimer, the minimum limiting distance for ET was the distance between the FMN1-HEME2 cross domains of neighbouring homodimer monomers, which was 34 Å, and gave the constant *k*_et_ = 0.0004 s^−1^, which gives a too low ET rate and contradicts the data in the literature, where the ET rate for this mutant is increased compared to WT. However, it should be noted that for the A83F form, the approach of the aromatic group F262 with heme is observed during dimerization from 5 Å to 3 Å, which can facilitate ET and increase *k*_cat_.

## 2. Results

### 2.1. Prediction of the Spatial Structure of the Full-Length CYP102A1/WT Monomer

**Figure 1:**
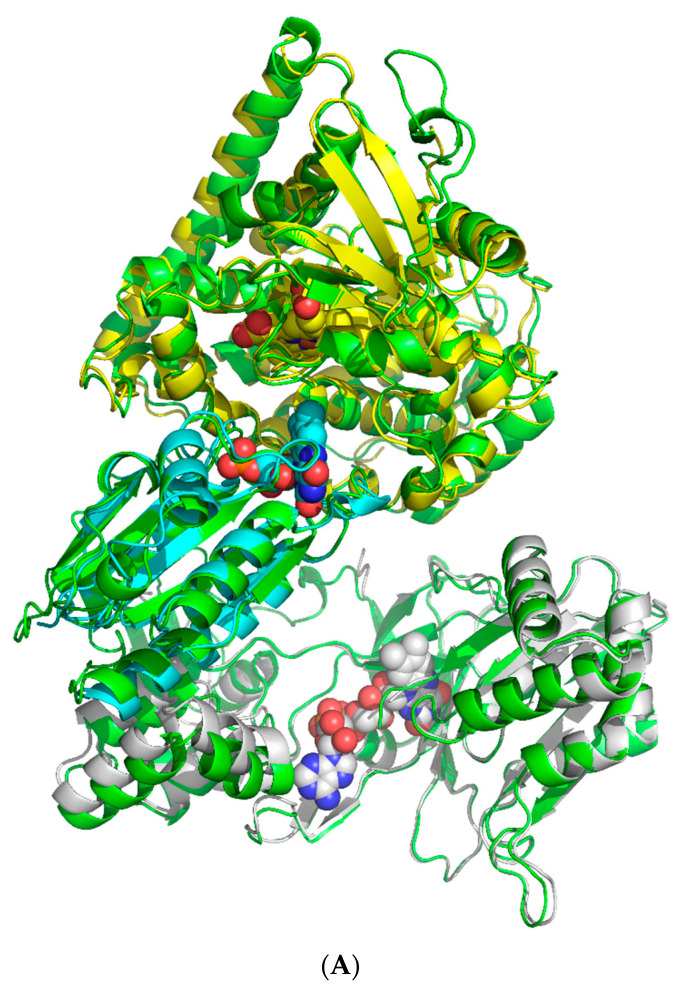

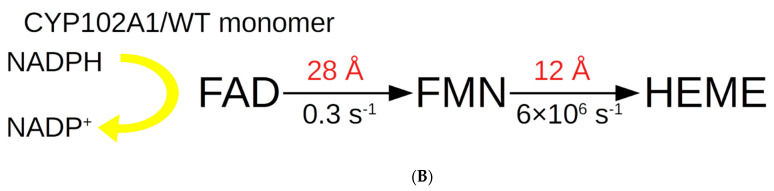
(**A**) Alignment of experimental structures of individual domains to predicted CYP102A1/WT structure (green): heme (PDB ID: 1BVY; yellow), FMN-domain (PDB ID: 1BVY; cyan) and FAD-domain (PDB ID: 4DQK; grey) on the structure of the CYP102A1/WT monomer obtained in AF2. Spheres show crystallised heme (pseudo colours, yellow carbon atoms), FMN (pseudo colours, cyan carbon atoms), FAD (pseudo colours, grey carbon atoms). (**B**) Schematic diagram of electron transport in the electron transport chain of the CYP102A1/WT monomer.

A prediction of the full length CYP102A1/WT in the AF2 program was made and compared with individual domains for which the experimental structure is known (1BVY for the heme and FMN domains; 4DKQ for the FAD domain [11]). The RMSD between the predicted monomer CYP102A1/WT and the heme domain from 1BVY was 0.6 Å, and with the FAD domain it was 0.7 Å. The prediction can be considered successful. Typical distances between prosthetic domain groups are shown in Figure 1. Hereafter, distances are assumed to be between flavogroups and heme.

### 2.2. Predicting CYP102A1A/WT Dimerization in AlphaFold Multimer

**Figure 2:**
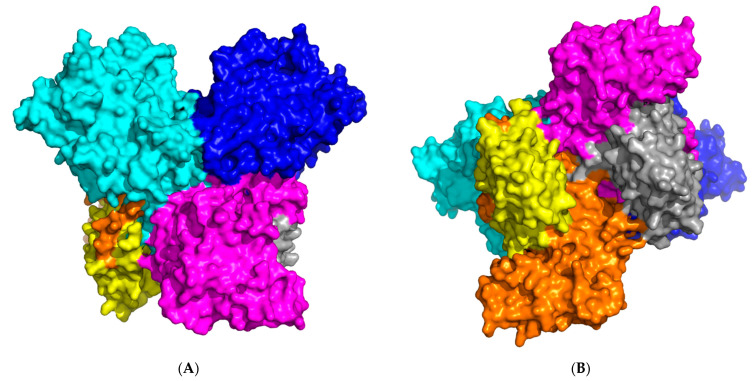

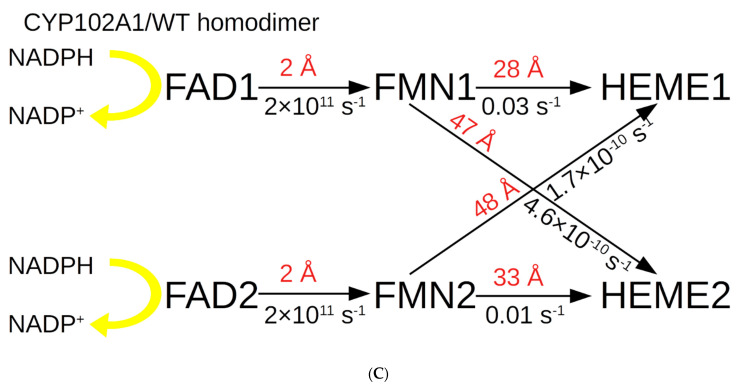
(**A**) Spatial structure of CYP102A1/WT homodimer, where (**A**,**B**) are two planes ((**B**) rotated relative to (**A**) 90 degrees down the horizontal axis) of the structure obtained in AFMultimer. Each of the domains is shown in colour: heme (cyan and blue), FMN (yellow and grey), FAD (orange and magenta). (**C**) Schematic diagram of electron transport in the electron transport chain of CYP102A1/WT monomer.

**Figure 3:**
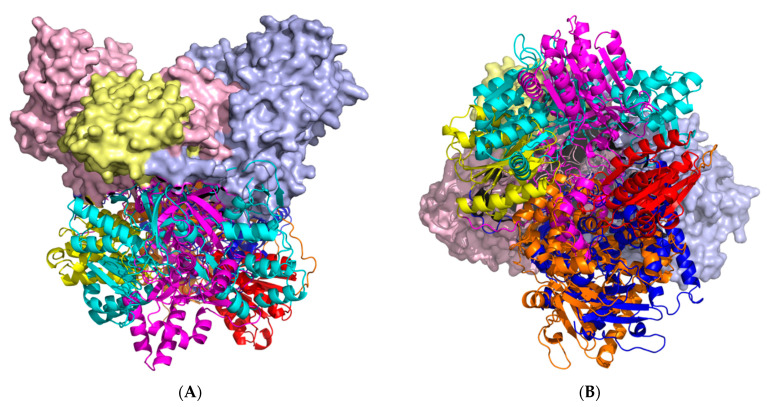
Alignment of monomer CYP102A1/WT predicted in AF2 to monomer from homodimer calculated using AFMultimer. The colours indicate the monomer domains: heme domain (orange), FMN domain (red), FAD domain (cyan). The surface denotes the second monomer of the homodimer, where light blue is heme domain 2, pale yellow is FMN-domain 2, light pink is FAD-domain 2. (**A**) shows a planar view of a superposition of 2 structures, where in (**B**) a downward rotation of 90 degrees along the horizontal axis is shown.

Further, taking into account that, according to experimental data, CYP102A1/WT can create homodimers, but the structure of these homodimers for the wild type is unknown, the structure of the dimer was predicted in an AlphaFold Multimer (AFMultimer). The AFMultimer predicted only one model, which was associated with the lack of similar experimental structures deposited in the Protein Data Bank (4DKQ for FAD-domain, 1BVY for FMN- and heme domains, respectively). As can be seen from Figure 2, AFMultimer predicts the existence of a homodimer in the form of a cross-structure of two monomers with asymmetric edges. Figure 3 shows that heme domains form complexes with each other and that FAD domains form complexes with each other. This is consistent with cryoEM data for the CYP102A1/A83F mutant form. The structures of the homotrimer and homotetramer were not predicted due to the high requirements for computing power which exceed the 60 GB RAM available. To carry out calculations of higher orders of oligomers, more efficient high-performance computing resources might be used in the future.

It can be seen that the limiting factor for the electron transfer constant from FAD to heme is the large distance of 30 Å between FAD and FMN. Calculations show that for the *k*_et_ between these PGs it is 0.03 s^−1^. It should be mentioned that *k*_cat_ in the hydroxylation of indole for this form is 0.03 s^−1^, which is in good agreement with the data on *k*_et_ for this form. In the case of cross-electron transport from FMN1 to HEME2 and from FMN2 to HEME1 between different monomers of the homodimer, the distance between these groups is 47 Å and 48 Å, respectively, which does not allow efficient implementation of ET between different monomeric units in the homodimer.

There are significant differences (RMSD 27.5 Å) between the monomer and monomer of the CYP102A1/WT homodimer, indicating significant conformational rearrangements that occur during dimerization (Figure 3). At the same time, the CYP102A1/WT domains, which have a globular structure, are consistent with the experimental structures of the heme, FMN, and FAD domains (RMSD = 0.8 Å, RMSD = 0.4 Å, RMSD = 0.6 Å, respectively).

### 2.3. Prediction of the Spatial Structure of the Full-Length CYP102A1/A83F and A83I Monomer

The full-size monomer CYP102A1/A83F and A83I was predicted in the AF2 program and compared with individual domains for which the experimental structure is known (i.e., WT and A83F). The visualised structures are shown in Supplementary Materials: Figures S1 and S2, respectively. In general, there is a high similarity of the structures of individual domains of mutants with the experimental structures of WT. To confirm this, a pairwise alignment of the wild-type WT and A83F heme domains was performed. The RMSD equaled 0.79 Å, which indicated a high similarity between the WT heme domain and the A83F mutant.

Pairwise alignment of CYP102A1/A83F and CYP102A1/A83I structures is poorly adjusted, as the RMSD was 22 Å. The main contribution to the RMSD growth is made by the discrepancy between the spatial positions of the FMN and FAD domains relative to the WT.

**Figure 4:**
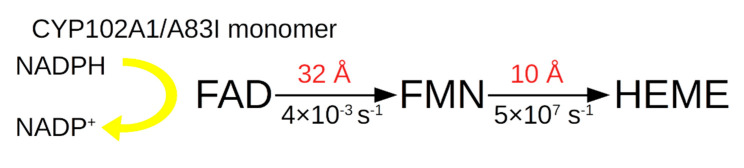
Scheme of electron transport in the electron transport chain of the CYP102A1/A83I monomer.

The situation with the electron transport scheme for the A83I mutant is considered below, taking into account the calculated distances (Figure 4).

It can be inferred that for A83I the distance limiting the ET between FAD and FMN is 32 Å, which provides *k*_et_ = 0.004 s^−1^, much less than *k*_cat_ = 0.19 s^−1^.

**Figure 5:**
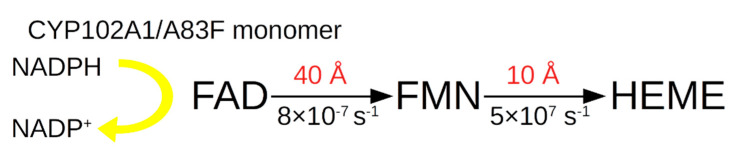
Scheme of electron transport in the electron transport chain of the CYP102A1/A83F monomer.

Next, the situation with the electron transport scheme for the A83F mutant is considered, taking into account the calculated distances. Below is a diagram of the electronic transport in this mutant (Figure 5).

As for A83F, it can be seen that the limiting factor for the electron transfer constant from FAD to heme is the large distance of 40 Å between FAD and FMN. Calculations show that for β = 1.06, *k*_et_ between these PG is 8 × 10^−7^ s^−1^. It should be mentioned that *k*_cat_ in the hydroxylation of indole for this form is 6 s^−1^, which is several orders of magnitude higher and does not agree with the data on *k*_et_ of this form.

### 2.4. Prediction of the Spatial Structure of Full-Length CYP102A1/A83I/A83F Homodimers

A prediction of the structure of homodimers CYP102A1/A83I and A83F was made in AFMultimer. Their spatial structures are presented in Figures S3 and S4, respectively.

Figures S3 and S4 show that heme domains form complexes with each other, as well as FAD domains which form complexes with each other too.

The RMSD between homodimers A83F and WT is 2.3 Å, which indicates an improvement in the coincidence structure compared to the individual monomerised form (which was described in the section above).

As for the mutant form of A83I, the RMSD between the A83I homodimer and WT is 6.4 Å, which indicates not very good agreement compared to the individual monomerised forms.

It should be noted that although there is an improvement in coincidence for the wild-type mutant forms, there is, however, a difference in the relative position of the spatial structures of FAD and FMN relative to the wild-type.

**Figure 6:**
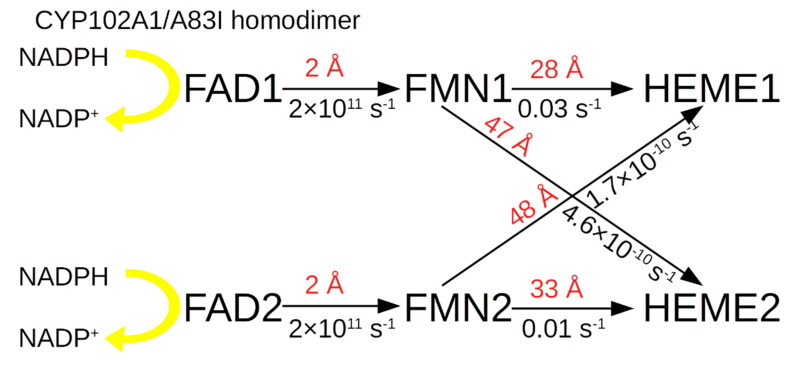
Schematic diagram of electron transport in the electron transport chain of the homodimer in CYP102A1/A83I.

ET schemes for the A83I mutant in the homodimer are presented below (Figure 6).

It can be inferred that for A83I the limiting factor for the electron transfer constant from FAD to heme is the large distance of 20 Å between FMN1 and HEME1. Calculations show that *k*_et_ between these PGs is 1 × 10^3^ s^−1^. It should be noted that *k*_cat_ in the hydroxylation of indole for this form is less than for the wild form and is 0.19 s^−1^.

In the case of cross electron transport from FMN1 to HEME2 and from FMN2 to HEME1 between different monomers of the homodimer, the distance between these groups is 43 Å and 45 Å, respectively, which does not allow efficient implementation of ET between different monomeric units in the homodimer, for which the ET rate is 3 × 10^−8^ and 4 × 10^−9^ s^−1^.

**Figure 7:**
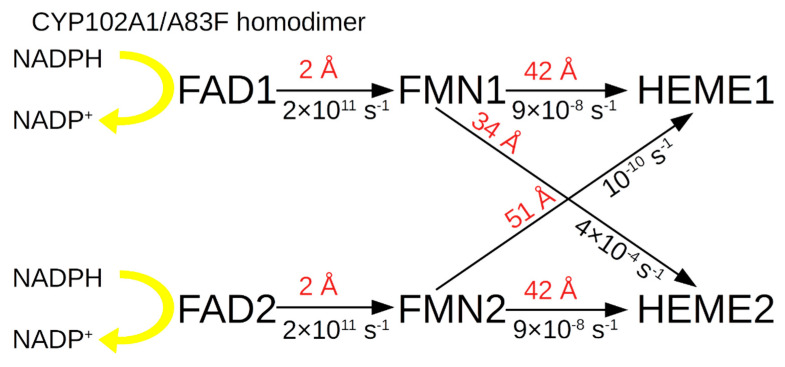
Scheme of electron transport in the electron transport chain of the homodimer in CYP102A1/A83F.

The diagram for the CYP102A1/A83F mutant is below (Figure 7).

For a homodimer of this form, the limiting distance is 34 Å between cross-domains FMN1→HEME2 of different monomers in the homodimer, and not between neighboring domains in the same homodimer monomer, which makes it possible to implement ET with *k*_et_ = 4 × 10^−4^ s^−1^, but with a reduced rate compared to WT and with an increased rate relative to the monomer of the mutant. According to the literature, for this mutant *k*_cat_ = 6 s^−1^, which is much higher than *k*_cat_ for WT (*k*_cat_ = 0.03 s^−1^).

It should be mentioned that for this form of protein during dimerization, a decrease in the interprotein distance between FMN1→HEME2 is observed, compared to the FMN→HEME distance in the monomer and, also, a decrease in the F262→HEME distance from 5 Å to 3 Å is observed, which possibly affects the rise of *k*_et_.

## 3. Discussion

In this work, the structures of monomers and homodimers of CYP102A1 and its mutants were calculated to identify possible pathways for efficient electron transport and the effect of mutations on this transport. As noted above, there is no information in the literature on the spatial structure of CYP102A1/WT with atomic resolution. In the presented work, the theoretical prediction of the CYP102A1/WT monomer and comparison with the two-domain truncated protein resolved by X-ray diffraction analysis was performed for the first time. The heme and FMN domains in the monomer were found to coincide with the experimental structure with RMSD = 0.6 Å for each domain. Thus, within the margin of error, AFMultimer prediction by truncated protein coincides with X-ray diffraction data. This suggests that the program is working successfully and can be applied to other proteins of the cytochrome P450 superfamily. This requires nothing but the knowledge of the primary amino acid sequence. The predicted structure should be checked with cryoEM data, but only a map of the EM density of the mutant CYP102A1/A83F form is available, not the wild type, and for the mutant form, the available EM map cannot be used by a wide range of researchers not working with cryoEM.

As for WT, it should be noted that the prediction of the structure of individual domains in the CYP102A1 monomer using the AF2 program is 0.6–0.8 Å; the coincidence of the dimer structure calculated by AFMultimer with the X-ray diffraction data is ~1 Å. This is at the level of determining the precise determination of the structure of the EM dimeric form of the protein in comparison with the experimental structure.

In this work, it was found that, despite the fact that the structures of individual flavin and heme domains, both in the monomer and in the dimer, coincide with the PDB-bank data at a level of 0.6 Å, in the homodimer monomer there is a rotation of one of the domains relative to the other in the region of the linker (indicate the AK linkers), connecting them at a significant angle with respect to their mutual structure in the dimer.

The distances between prosthetic groups for wild-type CYP102A1 as well as mutants were calculated and presented in the Results section.

Analysis of these distances enables revealing the effect of point mutations on the rate of electron transfer during hydroxylation, e.g., of indole. It is known that CYP102A1 is catalyzed by NADPH to FAD^+^, which is then transferred to FMN and then enters the heme, where the hydroxylation reaction takes place [4]. For all the three forms of proteins, there are data on the *k*_cat_ of the indole hydroxylation product.

The electron transport model should include consideration of all three domains, i.e., FAD, FMN and heme-containing. The distance between both FAD and FMN domains in the WT monomer is 28 Å, and the distance between FMN and heme is 11 Å. This means that the full path in the monomer between FAD and heme (FAD→FMN→HEME) is 39 Å. The electron transfer rate for such a distance is very low, and should be *k*_cat_ = 2 × 10^−6^ s^−1^, which is unrealistic, since for CYP102A1/WT *k*_cat_ for indole hydroxylation is 0.03 s^−1^.

Obviously, this approach is not correct enough, as it is necessary to take into account not only the full path of the electron between the donor and the acceptor, but also intermediate centers [10]. The ET scheme of this model for all forms of proteins is described below.

In monomerised forms of all proteins WT, A83F, A83I, the distances between FAD and FMN are 40–28 Å, respectively. They are the maximum distances in the ET chain in the monomers of these proteins. They are the limiting links in the electron transport chain in the protein globule.

Consider the *k*_et_ for the WT form monomer first. Based on formula (2), the *k*_et_ calculated in our work for β = 1.06 Å^−1^ is *k*_et_ = 0.3 s^−1^, which is much higher than that presented in the experimental data for which *k*_cat_ = 0.03 s^−1^ [9] This discrepancy might be due to the fact that *k*_cat_ for WT and mutants was measured at an enzyme concentration of C = 1 × 10^−7^–5 × 10^−7^ M. This means that the proportion of monomers in the mixture is about 20–8% depending from protein concentration and, accordingly, the contribution to the catalytic constant of hydroxylation of indole is made mainly by dimer molecules. The proportion was estimated based on the complex formation equation A + A ⇄ AA (where A is the concentration of CYP102A1/WT monomers) and the fact that the *K*_D_ of CYP102A1/WT dimerization is 1.1 nM [4], while neglecting the proportion of trimers and higher-order complexes for, and also assuming that the *K*_D_ of mutants and wild type are the same simplifying the estimation.

For the mutant form of the monomer A83I (*k*_cat_ (experimentally measured) value is 0.19 s^−1^) [9], which is higher than *k*_cat_ (experimentally measured) for WT (0.03 s^−1^); At the same time, for monomers of this mutant form, the distances (32 Å) between FAD and FMN are much higher than the corresponding values for WT (28 Å), which leads to a much lower *k*_et_, which should be 4 × 10^−3^ s^−1^.

For the A83F mutant, the calculated constant is 8 × 10^−7^ s^−1^ for the monomer, which is many orders lower than the experimental data.

The *k*_et_ estimates for protein dimers show the following:

For WT and mutant proteins, there are significant changes between the FMN→HEME prosthetic groups compared to the wild type in the dimer. The analysis showed that for the dimeric forms of proteins, both wild type and mutants, the FAD→FMN distance decreases to 2 Å, which is much less than the FMN and heme distance in the same protein isoforms.

Another feature noted is that the distances between the cross groups in the FMN1→HEME2, FMN2→HEME1 dimer are much larger than the FMN1→HEME1, FMN2→HEME2 distances for WT and A83F. Therefore, for these proteins, FMN1→HEME1 and FMN2→HEME2 are considered as the electron transport pathway of electrons based on the model of the tunnelling mechanism of electron transfer.

For the dimeric form of WT, the limiting stage is to overcome the distance between FMN1→HEME1, which is 30 Å. Calculations show that *k*_et_ is in the range of 0.03 s^−1^, which corresponds with the experimentally measured values *k*_cat_ = 0.03 s^−1^.

As for the A83I mutant homodimer, a decrease in the FMN2→HEME2 distance to 20 Å is observed in comparison with the monomeric form, which is much less than for the wild type. Calculations show that this results in *k*_et_ increase for this protein form compared to the wild type up to 10^3^ s^−1^. In the literature, *k*_cat_ for this mutant as compared to WT is noted to increase by six times, although it is not significant [9].

The situation with A83F is more difficult. For the dimer of this protein form, the limit is the distance between the FMN and the heme of the monomers in the homodimer, the minimum one is for FMN2→HEME2 and it equals 42 Å. For this distance, calculations show *k*_et_ = 9 × 10^−8^ s^−1^, which is much lower than the experimental value of 6 s^−1^. That is, in the calculation model for this mutant, not an increase but rather a decrease in the rate of intraprotein ET is observed. The explanation of this effect is as follows. First, between FMN1→HEME2 neighbouring monomers in a homodimer, a decrease in the distance to 34 nm is observed. Second, the replacement of A by F results in a decrease in the distance between F262 and heme to 3 Å, which in turn leads to a significant increase in the interactions between F262 and heme, and an additional decrease due to this barrier in the reaction of protein-protein ET in the homodimer. It should be noted that aromatic groups near the ET region can significantly increase the ET [12,13].

Thus, the structure of monomers and dimers of CYP102A1 calculated using AF2 and AFMultimer allows calculations of *k*_et_ in proteins as in WT.

## 4. Materials and Methods

The original CYP102A1/WT sequence was obtained from UniProt KB and the protein structures were obtained from the RCSB Protein Data Bank (Table 1). A series of cytochrome CYP102A1/WT/A83F and A83I predictions was performed for both monomers and homodimers in AF2 and AFM version 2.1.1 [7,8], respectively. For further analysis, structures with the best prediction quality were selected, if possible. Preparation of molecular graphics images and minimal distance measurements between atoms of PG was carried out in PyMol version 2.3.0 (Schrödinger, LLC, New York, NY, USA).

The calculation of *k*_et_ was carried out as follows. The data from the literature [9] on the rate of product formation as a result of the reaction of indole hydroxylation by the CYP102A1/WT enzyme were used. The rate equals *k*_cat_ = 0.03 s^−1^. The wild type CYP102A1 WT was chosen because it is the most studied of all the studied proteoforms of CYP102A1.

It was assumed that the rate of the hydroxylation product formation was limited by the rate of electron transport between the donor–acceptor groups FAD→FMN→HEME. According to the theory of the tunnel mechanism of electron transfer by Marcus, in the approximation of a nonadiabatic process, the dependence of the probability of electron transport on distance, which determines the rate constant of electron transfer, can be represented as Equation (2) [12].

Therefore, the values of β and A_0_ were obtained by solving a system of two equations of type (2) for the selected two pairs of parameters *k*_et_ = 10^10^ s^−1^ and R = 5 Å and *k*_et_ = 2.5 × 10^5^ s^−1^ and R = 15 Å for cytochrome, taking into account the fact that for proteins during electron transfer between metal-containing centers at a distance of R = 5–15 Å, *k*_et_ is observed in the range of 10^10^–10^5^ s^−1^ *k*_et_ = 2.5 × 10^5^ s^−1^ [13] and taking into account that *k*_et_ = 0.03 s^−1^ and R = 30 Å for the CYP102A1/WT homodimer, suggesting that under the conditions of the experiment carried out in [8], this enzyme was mainly in the form of homodimers (because *K*_D_ = 10^−9^ M [4]) at C = 0.1 μM. From where β equaling 1.06 Å^−1^ was determined.

## 5. Conclusions

In the present work, the prediction of the structural organization of the monomeric and homodimeric forms of CYP102A1/WT was carried out employing the AF2 and AFMultimer, respectively. Then a comparative analysis of the distances in these structures between the donor-acceptor groups (FAD→FMN→HEME) was carried out, from which the rate constants of electron transfer between these groups were calculated. A high level of agreement with the experimental structures of globular domains was shown, which was a good reason to the further use of AF2 and AFMultimer to predict the structure of the full-length monomeric protein and its homodimeric form of WT as well as A83I and A83F mutants. Problems arising during the interpretation of the AFMultimer results are under discussion. The obtained results of the distances between prosthetic groups in homodimers make it possible to use them to refine existing models and develop new models describing CYP102A1, which catalyze hydroxylation. For this, the theory of electron transport based on the tunneling mechanism with an exponential decrease in the rate of electron transport between the electron-acceptor groups FAD→FMN→HEME was used. A good agreement was obtained for *k*_et_ for the CYP102A1/WT dimeric form with the experimental data of the dimeric form on the rate of hydroxylation of this indole system; at the same time, *k*_et_ for the monomer was much lower than *k*_cat_, so that the hydroxylation reaction should proceed with the participation of the dimeric form of WT. For mutant forms of CYP102A1/A83I, the calculations also show an increase in *k*_et_ as does the literature, which reflects a trend towards an increase in the activity of this form, although to a greater extent than observed in the experiment. For CYP102A1/A83F, the calculation of *k*_et_ should account for the contribution of the F262 aromatic group, which shifts closer to heme, as predicted by AFMultimer for the dimeric form.

## Figures and Tables

**Table 1:** Information about the structures of the studied protein molecules.

Domain	UniProt ID	PDB ID	Amino Acid Sequence Length	References
HEME WT	P14779	1BVY	2–459	[4]
FMN WT			460–650	
FAD		4DQK	659–1049	[11]

## Data Availability

Not applicable.

## Supplementary Materials

The following supporting information can be downloaded. Figure S1: Alignment of experimental structures of individual domains to predicted CYP102A1/A83I structure; Figure S2: Alignment of experimental structures of individual domains to predicted CYP102A1/A83F structure; Figure S3: Spatial structure of CYP102A1/A83I homodimer; Figure S4: Spatial structure of CYP102A1/A83F homodimer; Table S1: Minimal distances between atoms of prosthetic groups (Å) and controlling constants (s^−1^). *k*_cat_ values are from literature data. The predicted structures of all proteins are presented in Protein Data Bank (.pdb) file format.

## References

[1] Archakov A.I., Bachmanova G.I. (1990). Cytochrome P-450 and Active Oxygen.

[2] Munro A.W., Girvan H.M., McLean K.J. (2007). Cytochrome P450--redox partner fusion enzymes. Biochim. Biophys. Acta.

[3] Su M., Chakraborty S., Osawa Y., Zhang H. (2020). Cryo-EM reveals the architecture of the dimeric cytochrome P450 CYP102A1 enzyme and conformational changes required for redox partner recognition. J. Biol. Chem..

[4] Neeli R., Girvan H.M., Lawrence A., Warren M.J., Leys D., Scrutton N.S., Munro A.W. (2005). The dimeric form of flavocytochrome P450 BM3 is catalytically functional as a fatty acid hydroxylase. FEBS Lett..

[5] Sevrioukova I.F., Li H., Zhang H., Peterson J.A., Poulos T.L. (1999). Structure of a cytochrome P450–redox partner electron-transfer complex. Proc. Natl. Acad. Sci. USA.

[6] Yip K.M., Fischer N., Paknia E., Chari A., Stark H. (2020). Atomic-resolution protein structure determination by cryo-EM. Nature.

[7] Jumper J., Evans R., Pritzel A., Green T., Figurnov M., Ronneberger O., Tunyasuvunakool K., Bates R., Žídek A., Potapenko A. (2021). Highly accurate protein structure prediction with AlphaFold. Nature.

[8] Evans R., O’Neill M., Pritzel A., Antropova N., Senior A., Green T., Žídek A., Bates R., Blackwell S., Yim J. (2021). Protein complex prediction with AlphaFold-Multimer. BioRxiv.

[9] Huang W.C., Westlake A.C.G., Maréchal J.D., Joyce M.G., Moody P.C.E., Roberts G.C.K. (2007). Filling a hole in cytochrome P450 BM3 improves substrate binding and catalytic efficiency. J. Mol. Biol..

[10] Rubin A.B. (1994). Lectures on Biophysics.

[11] Joyce M.G., Ekanem I.S., Roitel O., Dunford A.J., Neeli R., Girvan H.M., Baker G.J., Curtis R.A., Munro A.W., Leys D. (2012). The crystal structure of the FAD/NADPH-binding domain of flavocytochrome P450 BM3. FEBS J..

[12] Bollinger M.J. (2008). Electron relay in proteins. Science.

[13] Shih C., Museth A.K., Abrahamsson M., Blanco-Rodriguez A.M., Di Bilio A.J., Sudhamsu J., Crane B.R., Ronayne K.L., Towrie M., Vlček A. (2008). Tryptophan-accelerated electron flow through proteins. Science.

