# Supplementary material for: Prediction of Monomeric and Dimeric Structures of CYP102A1 Using AlphaFold2 and AlphaFold Multimer and Assessment of Point Mutation Effect on the Efficiency of Intra- and Interprotein Electron Transfer
Source: Molecules. 2022 Feb 18;27(4):1386. doi: 10.3390/molecules27041386 (PMC8874714; doi:10.3390/molecules27041386)
Supplement: Supplementary file 1 [file molecules-27-01386-s001.zip › Suppl_Figures.pdf]

Supplementary Information

Prediction of monomeric and dimeric structures of CYP102A1 using AlphaFold2 and AlphaFold Multimer and assessment of point mutation effect on the efficiency of intra- and interprotein electron transfer

Yuri D. Ivanov [1,2], Taldaev Amir [1,3*], Andrey V. Lisitsa [1], Elena A. Ponomarenko [1], Alexander I. Archakov [1]

[1] Laboratory of Nanobiotechnology, Institute of Biomedical Chemistry, Pogodinskaya St. 10/8, 119121 Moscow, Russia

[2] Laboratory of Shock Wave Impacts, Joint Institute for High Temperatures of the Russian Academy of Sciences, Izhorskaya St. 13 Bd.2, 125412 Moscow, Russia

[3] Department of Chemistry, Sechenov First Moscow State Medical University (Sechenov University), Trubetskaya St. 8/2, 119991 Moscow, Russia

* Correspondence: t-amir@bk.ru

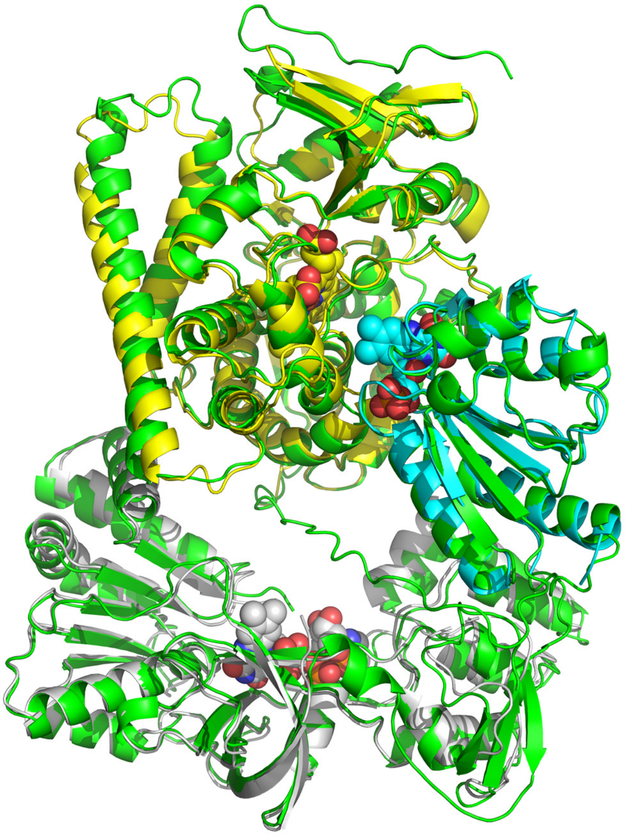

Supplementary Figure S1. Alignment of experimental structures of individual domains to predicted CYP102A1/A83I structure (green): heme (PDB ID: 1BVY; yellow), FMN-domain (PDB ID: 1BVY; cyan) and FAD-domain (PDB ID: 4DQK; grey) on the structure of the CYP102A1/WT monomer obtained in AF2. Spheres show crystallized heme (pseudo colors, yellow carbon atoms), FMN (pseudo colors, cyan carbon atoms), FAD (pseudo colors, grey carbon atoms).

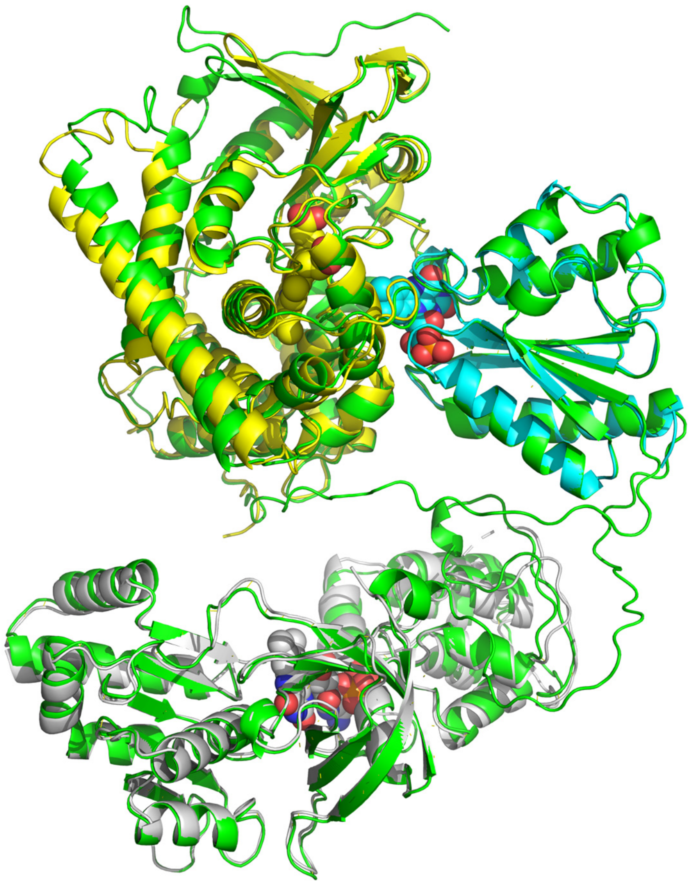

Supplementary Figure S2. Alignment of experimental structures of individual domains to predicted CYP102A1/A83F structure (green): heme (PDB ID: 1BVY; yellow), FMN-domain (PDB ID: 1BVY; cyan) and FAD-domain (PDB ID: 4DQK; grey) on the structure of the CYP102A1/WT monomer obtained in AF2. Spheres show crystallized heme (pseudo colors, yellow carbon atoms), FMN (pseudo colors, cyan carbon atoms), FAD (pseudo colors, grey carbon atoms).

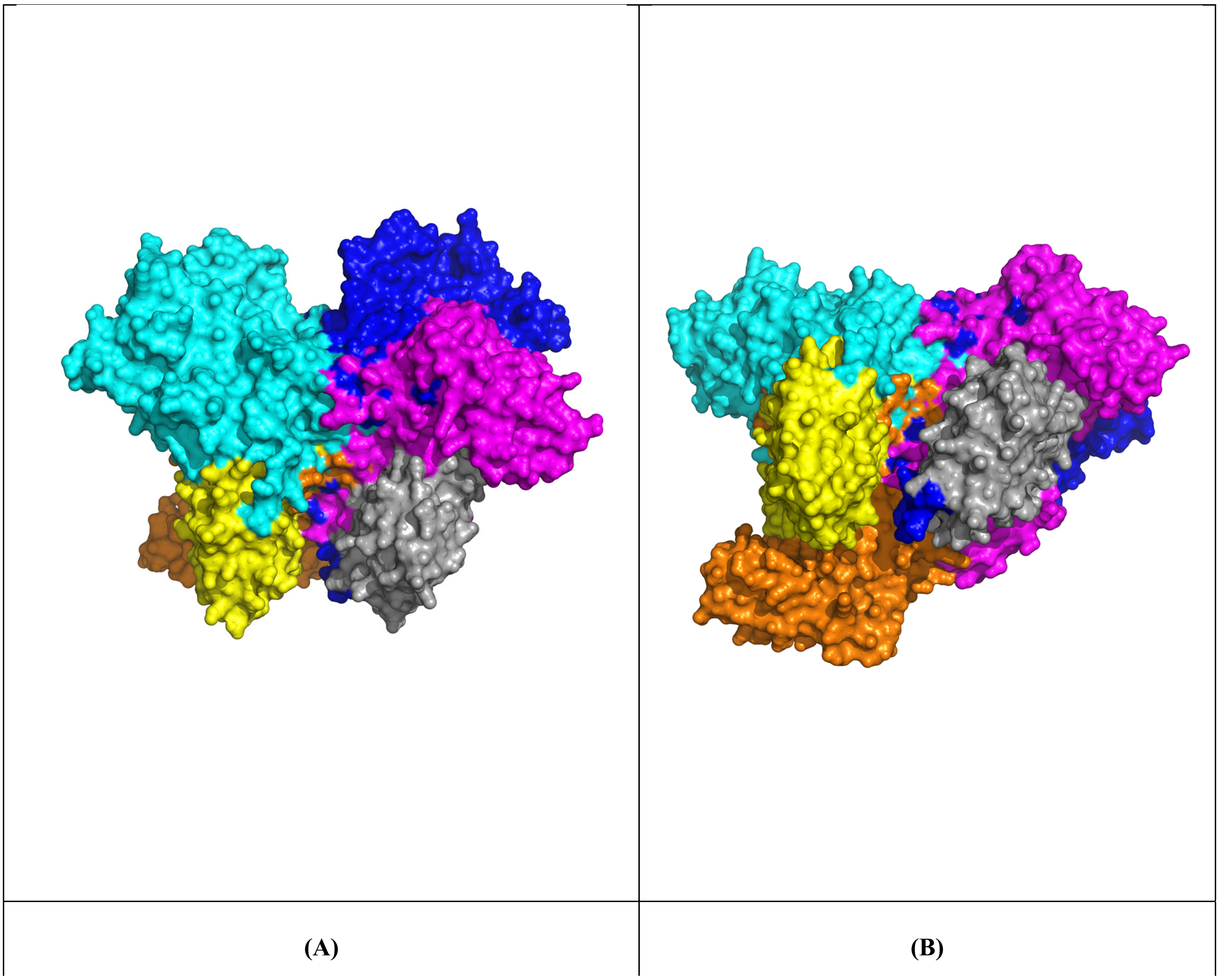

Supplementary Figure S3. Spatial structure of CYP102A1/A83I homodimer, where Figures S3A and S3B are two planes (Figure S3B rotated relative to S3A 90 degrees down the horizontal axis) of the structure obtained in AFMultimer. Each of the domains is shown in color: heme (cyan and blue), FMN (yellow and grey), FAD (orange and magenta).

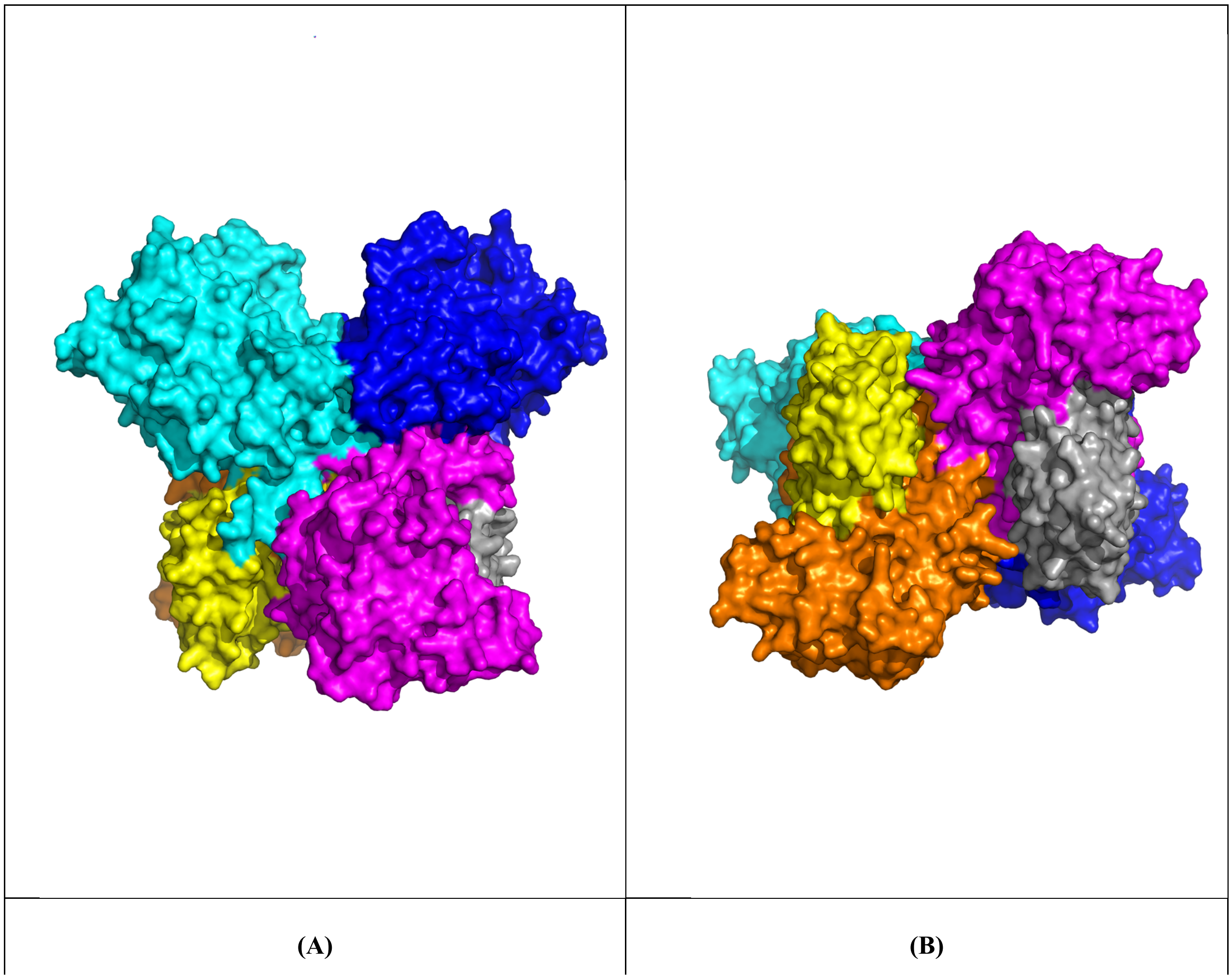

(A) (B)

Supplementary Figure S4. Spatial structure of CYP102A1/A83F homodimer, where Figures S4A and S4B are two planes (Figure S4B rotated relative to S4A 90 degrees down the horizontal axis) of the structure obtained in AFMultimer. Each of the domains is shown in color: heme (cyan and blue), FMN (yellow and grey), FAD (orange and magenta).

Table S1. Minimal distances between atoms of prosthetic groups (Å) and controlling constants (s^{-1}). k_{cat} values are from literature data [].

**Distances between prosthetic groups**	**Experimental CYP102A1/WT structure (PDB ID: 1BVY)**	**AF2 CYP102A1/WT monomer prediction**	**AFMultimer CYP102A1/WT homodimer prediction**	**AF2 CYP102A1/A83F monomer prediction**	**AFMultimer CYP102A1/A83F homodimer prediction**	**AF2 CYP102A1/A83I monomer prediction**	**AFMultimer CYP102A1/A83I homodimer prediction**
FAD1-FMN1	-	28 (k_{et}=0.3; k_{cat}=0.03)	2	40 (k_{et}=8*10^{-7}; k_{cat}=6)	2	32 (k_{et}=0.01; k_{cat}=0.03)	2
FMN1-HEME1	14	11	30 (k_{et}=0.03; k_{cat}=0.03)	10	42 (k_{et}=0.01; k_{cat}=6)	10	28 (k_{et}=0.01; k_{cat}=0.03)
FAD2-FMN2	-	-	2	-	2	-	2
FMN2-HEME2	-	-	33 (k_{et}=0.01; k_{cat}=0.03)	-	42 (k_{et}=0.01; k_{cat}=6)	-	20 (k_{et}=0.01; k_{cat}=0.03)
FMN1-HEME2	61	-	48	-	34 (k_{et}=0.01; k_{cat}=6)	-	43 (k_{et}=0.01; k_{cat}=0.03)
FMN2-HEME1	-	-	47	-	51 (k_{et}=0.01; k_{cat}=6)	-	45 (k_{et}=0.01; k_{cat}=0.03)
FAD1-FAD2	-	-	37	-	40	-	32
RMSD exp. - HEME1	-	0.61	0.75	0.66	0.78	0.73	0.78
RMSD exp. - HEME2	-	-	0.66	-	0.67	-	0.66
RMSD exp. - FMN1	-	0.59	0.47	0.40	0.47	0.48	0.50
RMSD exp. - FMN2	-	-	0.47	-	0.47	-	0.47
RMSD exp. - FAD1	-	0.61	0.68	0.71	0.72	0.86	0.72
RMSD exp. - FAD2	-	-	0.68	-	0.72	-	0.75
